# Transforming growth factor-β increases interleukin-13 synthesis via GATA-3 transcription factor in T-lymphocytes from patients with systemic sclerosis

**DOI:** 10.1186/s13075-015-0708-0

**Published:** 2015-07-31

**Authors:** Julie Baraut, Dominique Farge, Francette Jean-Louis, Ingrid Masse, Elena Ivan Grigore, Lucas C. M. Arruda, Jérôme Lamartine, Franck Verrecchia, Laurence Michel

**Affiliations:** INSERM U976, Pavillon Bazin, Hôpital Saint-Louis 1, Avenue Claude Vellefaux, 75010 Paris, France; Unité Clinique de Médecine Interne et Pathologie Vasculaire, UF 04, Hôpital Saint-Louis, AP-HP Assistance Publique des Hôpitaux de Paris, INSERM UMRS 1160, Paris, France; Université Claude Bernard Lyon I et CNRS UMR5534, Centre de Génétique et de Physiologie Moléculaire et Cellulaire, Villeurbanne, F-69622 France; Department of Biochemistry and Immunology, Ribeirão Preto Medical School, University of São Paulo, Ribeirão Preto, Brazil; Center for Cell-based Therapy, São Paulo Research Foundation (FAPESP), São Paulo, Brazil; INSERM U957, Université de Nantes, 1 rue Gaston Veil, 44000 Nantes, France

## Abstract

**Introduction:**

Transforming growth factor (TGF)-β and interleukin (IL)-13 play a crucial role in the pathogenesis of systemic sclerosis (SSc), partly through activation of collagen production that leads to fibrosis. The aim of the present study was to determine whether TFG-β alters IL-13 production in T lymphocytes from patients with SSc from that seen in those of healthy donors.

**Methods:**

IL-13 mRNA and protein synthesis under TFG-β exposure was measured in circulating T lymphocytes from healthy donors and patients with SSc and also in the Jurkat Th2 T-cell line, using quantitative real-time PCR and fluorescence-activated cell sorting analysis, respectively. The involvement of Smad and GATA-3 transcription factors was assessed by using specific inhibitors and small interfering RNA, and the binding capacity of GATA-3 to the IL-13 gene promoter was evaluated by chromatin immunoprecipitation assay.

**Results:**

TGF-β induced a significant decrease in IL-13 mRNA and protein levels in lymphocytes from healthy donors (mean [±SD] inhibition of 30 % ± 10 % and 20 % ± 7 %, respectively; *p* < 0.05). In contrast, TGF-β promoted a significant increase in IL-13 mRNA levels and IL-13 synthesis by CD4^+^ and CD8^+^ T-cell subtypes from patients with SSc, with respective increases of 2.4 ± 0.3-fold, 1.6 ± 0.05-fold and 2.7 ± 0.02-fold. The involvement of the Smad signaling pathway and upregulation of GATA-3 binding capacity on the IL-13 promoter in lymphocytes from patients with SSc contributed to the effect of TGF-β on IL-13 production.

**Conclusions:**

These results demonstrate that TGF-β upregulates IL-13 synthesis through GATA-3 expression in the T lymphocytes of patients with SSc, confirming that the GATA-3 transcription factor can be regarded as a novel therapeutic target in patients with SSc.

## Introduction

Systemic sclerosis (SSc) is a systemic autoimmune and inflammatory disease with a heterogeneous clinical presentation characterized by progressive fibrosis of the skin and internal organs, vascular damage, autoantibody production and immune dysregulation [1, 2]. Genome-wide transcription profiles of skin biopsy specimens obtained from patients with scleroderma have provided direct evidence of the involvement of cytokines in the inflammatory and fibrotic processes of SSc [3, 4]. Particularly, elevated levels of two type 2 helper (Th2) T-cell–produced cytokines (transforming growth factor [TGF]-β and interleukin [IL]-13) have been observed in the serum and tissue of patients with SSc [5, 6], and these cytokines have been shown to be able to induce skin fibrosis [7–10].

TGF-β belongs to the TGF-β superfamily containing three homologous isoforms in mammals (TGF-β1, TGF-β2 and TGF-β3) encoded by different genes [11]. TGF-β1 is the predominant isoform, mainly expressed by circulating monocytes and tissue macrophages in the immune system [12]. The TGF-β1 signaling pathway mediates its biological functions via binding to type II TGF-β transmembrane receptor (TGFβRII). TGFβRII recruits and phosphorylates the type I receptor TGFβRI, which belongs to the activin receptor-like kinase (ALK) family, leading to cell signaling initiation via ALK5 involvement [11]. Upon phosphorylation by ALK5, receptor-regulated Smads R-Smad2 and R-Smad3 associate with co-Smad4 and translocate into the nucleus to bind to Smad-binding element in association with a large number of other transcription factors [13, 14]. This leads to transcriptional regulation of target genes, including those that encode extracellular matrix (ECM) proteins such as collagens I and III [15–17]. Besides the Smad canonical pathway, TGF-β1 is also able to activate Smad-independent signaling pathways, such as Ras- extracellular signal-regulated kinase (ERK), c-Jun N-terminal kinase (JNK), p38, mitogen-activated protein kinases (MAPKs) and phosphatidylinositol 3-kinase-Akt [15, 16, 18, 19]. In addition, TGF-β1 is one of the most potent profibrotic cytokines able to upregulate ECM proteins, downregulate matrix metalloproteinases (MMPs), induce myofibroblast differentiation and modulate the expression of various cytokine receptors, including its own [14]. The literature contains evidence that activation of the p38 pathway downstream to TGF-β receptors is also involved in the regulation of Th2 cytokine synthesis, including IL-4 and IL-13 [20, 21]. Indeed, overexpression of TGF-β1–dependent genes has been observed in biopsy specimens of skin lesions from patients with scleroderma [22].

TGF-β1 is also a potent immunomodulator, regulating T-cell proliferation, differentiation and survival in healthy humans [12, 23] by downregulating IL-2 transcription via Smad3 [24] or by directly targeting cell cycle regulators such as cyclin-dependent kinase inhibitors (p15, p27 or p21), c-Myc, cyclin D2 and cyclin E [25]. TGF-β1 can also inhibit Th1 and Th2 differentiation by repressing expression of the T-bet [26] and GATA-3 [27] transcription factors, respectively. It also induces FoxP3-expressing CD4^+^CD25^+^ regulatory T cells to indirectly influence T-cell activation [28].

Besides TGF-β, other crucial fibrosis inductors have been described, including IL-13 and IL-4. Both are produced by activated Th2 T cells and share many functional activities by using the same IL-4 receptor α-chain [29, 30]. Inhibition of IL-4 and IL-13 in an independent way identified IL-13 as the dominant effector cytokine of fibrosis [31]. IL-13 can promote tissue fibrosis by direct fibroblast activation, as well as by indirect pathways, via TGF-β production stimulation. IL-13 binds the IL-13 receptor α-chain 2 (IL-13Rα2) that induces upregulation of TGF-β promoter activities and uses activator protein AP-1 as a signaling pathway distinct from the one used by IL-4, leading to an increase in its own fibrogenic potential [9, 32]. Moreover, IL-13 induces the production of latent TGF-β1 from macrophages and TGF-β1 activation through upregulation of MMP9 expression, leading to lung fibrosis [9]. A fibrogenesis pathway that is IL-13–dependent but TGF-β1–independent has also been reported [33]. The importance of IL-13 in SSc susceptibility has been shown by genetic studies involving polymorphisms in the IL-13 [34] and IL-13Rα2 [35] genes, and many studies support the role of IL-13 in SSc pathogenesis [36–39]. Recently, Fuschiotti et al. showed that IL-13–producing CD8^+^ T cells are directly involved in modulating dermal fibrosis in SSc [10], confirming the importance of IL-13 in the pathogenesis of SSc dermal fibrosis.

Upregulation of IL-13 production with increased activation of GATA-3 has been shown in naïve CD8^+^ T cells from patients with SSc [40]. GATA-3 is the dominant transcription factor able to regulate Th2 differentiation and IL-13 production by Th2 effector T cells [41], and, although GATA-3 can be regulated by TGF-β, IL-13 regulation by TGF-β has not been completely studied in SSc.

We designed the present study to analyze whether the multifunctional growth factor TGF-β1 is able to modulate IL-13 synthesis in T cells and which signaling pathway is involved by using specific inhibitors of the TGF-β receptor kinase ALK5 at the top of the signaling pathway as well as the Smad and p38-MAPK pathways. Our results show that TGF-β significantly upregulates IL-13 synthesis in T lymphocytes from patients with SSc, whereas it acts as a negative modulator of IL-13 synthesis in T lymphocytes from healthy donors, via respective up- and downregulation of GATA-3 transcription factor activity.

## Methods

### Study subjects

Sixteen patients (eleven females) with diffuse cutaneous SSc were recruited from the Internal Medicine and Vascular Disease Unit of St. Louis Hospital (Paris, France). All patients fulfilled the classification criteria for SSc proposed by the American College of Rheumatology [42] and were included before having received any treatment. At the time of sampling, their median age was 48.5 (range, 25–78) years and median modified Rodnan skin score was 29.5 (range, 14–51). Blood samples from 12 healthy volunteers (6 females) from the Etablissement Français du Sang were used as controls (median [range] age, 34 (24–55) years). Patients and healthy donors gave their written informed consent after the study received approval by the local ethics committee. Because of the limitations of blood sample availability, some of the experiments were performed using T lymphocytes from only some of the patients with SSc or some of the healthy volunteers, as indicated. The Jurkat Th2 T-cell line (clone E6-1) was purchased from the European Collection of Animal Cell Cultures (Salisbury, UK).

### Cell cultures

Peripheral blood mononuclear cells (PBMCs) were isolated by Ficoll-Hypaque centrifugation (Amersham Pharmacia Biotech, Little Chalfont, UK), and peripheral blood lymphocytes (PBLs) were isolated from PBMCs by elimination of monocytes by adhesion for 2 h. All cultures were grown at 37 °C with 5 % CO_2_ at 0.2 × 10^6^ cells/ml in complete RPMI 1640 medium (Gibco; Life Technologies, Carlsbad, CA, USA) supplemented with 10 % fetal calf serum (FCS), 1 % penicillin-streptomycin and 1 % N-2-hydroxyethylpiperazine-N-2-ethanesulfonic acid (HEPES).

### Study design

PBLs from patients with SSc and healthy donors were cultured for 5 days on plates previously coated with anti-CD3 (clone HIT3a, 2 μg/ml; BD Pharmingen, San Diego, CA, USA) antibody at the concentration of 0.5 × 10^6^ cells/ml in complete RPMI 1640 medium supplemented with soluble anti-CD28 (clone CD28.2, 1 μg/ml; BD Pharmingen) antibody and IL-2 (20 U/ml; PeproTech, Rocky Hill, NJ, USA). At the sixth day, cells were stimulated for the last 4 h with a combination of phorbol 12-myristate 13-acetate (10 μg/ml; Sigma-Aldrich, St. Louis, MO, USA) and ionomycin (1 μM; Sigma-Aldrich) in the presence or absence of the optimal concentration of 5 ng/ml TGF-β1 (PeproTech), named *TGF-β* hereafter, in 0.5 % FCS-containing RPMI 1640 medium with or without a 1-h preincubation with specific inhibitors (SB431542 from Sigma-Aldrich; SB203580 or SIS3 from Calbiochem, San Diego, CA, USA). Jurkat T cells were cultured in 0.5 % FCS-containing medium for 16 h before addition of 5 ng/ml TGF-β for 30 min or 4 h.

### Flow cytometry

IL-13 production was determined by intracellular staining using phycoerythrin (PE)-labeled anti-human IL-13 antibody (clone JES10-5A2; BD Biosciences, San Jose, CA, USA). HiCK-2 human cytokine positive control cells (BD Pharmingen) were used as a positive control for IL-13 staining. Cell phenotype was assessed by staining with specific association of fluorescein isothiocyanate (FITC)-CD4, allophycocyanin (APC)-CD3 and PE-IL-13 antibodies or association of FITC-CD8, APC-CD3 and PE-IL-13 antibodies (all from BD Biosciences). Antibody isotypes (BD Biosciences) were selected to match these specific antibodies. Before intracellular staining, cells were incubated with BD GolgiStop (BD Pharmingen) for the last 4 h of stimulation, then fixed for 1 h at 4 °C in phosphate-buffered saline (PBS) containing 0.45 % formaldehyde before permeabilization for 15 min at 37 °C in PBS containing 0.2 % Tween-20. After two PBS washes, cells were incubated with PE isotype or PE-IL-13 antibodies at optimal concentrations in PBS for 30 min at 4 °C in the dark and then washed in PBS with 2 % FCS. Cells were next incubated with FITC or APC isotypes or FITC-CD4 or FITC-CD8 and APC-CD3 antibodies for membrane staining for 20 min at 4 °C in the dark and finally fixed with 1 % formaldehyde. Surface and intracellular expression was quantified using a FACSCalibur flow cytometer (BD Biosciences) with gate established on forward scatter and side scatter lymphocyte areas. Unstained cells or cells stained with isotype-matched antibodies were used to indicate nonspecific signals and establish the positive limits. Data were analyzed with Kaluza software (Beckman Coulter, Brea, CA, USA).

### Quantitative RT-PCR

Total RNA was extracted using an RNeasy™ Mini Kit (Qiagen, Hilden, Germany) according to the manufacturer’s instructions. DNase I treatment (25 U, 15 min) of total RNA was performed to eliminate genomic contamination of the RNA samples. One microgram of total RNA was used for first-strand cDNA synthesis using a RT-PCR kit (Invitrogen, Carlsbad, CA, USA) according to the manufacturer’s instructions. RT-PCR was performed with an ABI PRISM 7300 instrument (Applied Biosystems, Foster City, CA, USA) using SYBR Green PCR core reagents (Applied Biosystems). The β-glucuronidase (GUS) housekeeping gene expression was used as reference to normalize mRNA levels for each sample. The sequence of the forward primer for IL-13 mRNA was 5′-CGAGAAGACCCAGAGGATGCT-3′, and that of the reverse primer was 5′-ACTGCCCAGCTGAGACCTTGT-3′. For TGF-β mRNA, the forward primer was 5′- GGGAAATTGAGGGCTTTCG-3′ and the reverse primer was 5′- GAACCCGTTGATGTCCACTTG-3′. For GATA-3 mRNA, the forward primer was 5′- TGCGGGCTCTATCACAAAATG-3′ and the reverse primer was 5′- GCCTTCGCTTGGGCTTAAT-3′. The forward primer for GUS mRNA was 5′- GAAAATATGTGGTTGGAGAGCTCATT-3′ and the reverse primer was 5′- CCGAGTGAAGATCCCCTTTTTA-3′. The conditions for the one-step RT-PCR were as follows: 5 min at 95 °C, then 35 cycles of amplification at 95 °C for 30 s and 30 s at 55 °C, and finally 1 min at 72 °C and 10 min at 72 °C. Each assay was run in duplicate. All samples were normalized to GUS. Quantification of the target gene expression was done using the comparative cycle threshold (C_t_) method according to the manufacturer’s instructions (Applied Biosystems). An average C_t_ was calculated for the duplicate reactions and normalized to housekeeping gene GUS (ΔC_t_ = C_t_ sample − C_t_ GUS).

### RNA stability experiments

Jurkat T cells (5 × 10^6^) were stimulated with TGF-β for 4 h, followed by the addition of actinomycin D (3 μg/ml) to halt ongoing transcription. After 1, 3 and 5 h, cells were pelleted and total RNA was extracted using RNeasy™ Mini Kit for further quantification of IL-13 and GUS mRNA levels by quantitative RT-PCR (qRT-PCR).

### Transient cell transfections and reporter assays

Two million Jurkat T cells were electroporated with 2 μg of pGL3 2666-bp IL-13 promoter construct/Luciferase (2666 bp-IL13-Lux) or 2 μg of (CAGA)9-Lux/Luciferase (cytomegalovirus [CMV] Smad3/4-specific reporter construct [43]), using the Cell Line Nucleofector Kit (Lonza, Cologne, Germany). Empty pGL3- and CMV-Lux plasmids (2 μg) were transfected as respective controls. Transfection efficiency was estimated to 50–60 % by using cotransfection of a green fluorescent protein expression vector and cytometric analysis (data not shown). Firefly and Renilla luciferase activities were used for normalization using the Dual-Luciferase Reporter Assay System and a GloMax 20/20 luminometer (Promega, Madison, WI, USA).

### Western blot analysis

Nuclear and cytoplasmic protein extracts were isolated using a small-scale preparation (Promega) and stored at −80 °C until use. The protein concentration in the cytoplasmic or nuclear extracts was determined using a Bradford assay (Bio-Rad Laboratories, Hercules, CA, USA). Fifty micrograms of protein extracts were denatured by heating at 95 °C for 3 min before resolution by SDS-PAGE, electrotransferred to Hybond enhanced chemiluminescence nitrocellulose filters (Amersham Biosciences, Little Chalfont, UK) and immunoblotted with either anti–phospho-Smad3 (clone EP823Y) and anti-Smad3 (clone EP568Y) antibodies from EMD Millipore (Billerica, MA, USA), anti-GATA-3 (clone HG3-31) or anti-β-actin antibodies from Santa Cruz Biotechnology (Santa Cruz, CA, USA), all at 1:1000 concentration in PBS/5 % nonfat milk, for 1 h. After washing, filters were incubated with horseradish peroxidase–conjugated anti-rabbit or anti-mouse secondary antibodies (Santa Cruz Biotechnology). An enhanced chemiluminescence system (Amersham Biosciences) was used for detection. Equal protein loading was confirmed by using monoclonal β-actin blots (Sigma-Aldrich). Densitometry was done using the Imager FX System (Bio-Rad Laboratories) and analyzed using ImageJ software (National Institutes of Health, Bethesda, MD, USA).

### Chromatin immunoprecipitation

After fixation in 1 % formaldehyde, T cells (1 × 10^7^) were lysed for 5 min in 50 mM Tris buffer (pH 8) containing 0.2 mM ethylenediaminetetraacetic acid (EDTA), 0.1 % Nonidet P (NP)-40 and 10 % glycerol supplemented with anti-protease cocktail (Roche Life Science, Indianapolis, IN, USA). Nuclei were resuspended in 50 mM Tris buffer with 1 % SDS and 5 mM EDTA. Chromatin was sheared by sonication. After preclearing with protein A beads (Santa Cruz Biotechnology), lysates were incubated overnight at 4 °C with 1 μg/ml anti-GATA-3 (sc-269) or mouse immunoglobulin G (IgG) antibodies (Santa Cruz Biotechnology). Immune complexes were collected with protein A, washed three times with high-salt buffer (50 mM HEPES-KOH, 140 mM NaCl, 1 mM EDTA, 1 % Triton X-100, 0.1 % sodium deoxycholate), twice with low-salt buffer (10 mM Tris–HCl, 250 mM LiCl, 1 mM EDTA, 0.5 % NP-40, 0.1 % sodium deoxycholate) and then twice with Tris-EDTA (TE). Immune complexes were extracted in 1× TE buffer, and protein crosslinking was reverted by heating at 65 °C for 5 h. DNA was then extracted by phenol-chloroform and precipitated by ethanol, and a 1/20 fraction of the immunoprecipitated DNA was used for qRT-PCR.

### Statistical analysis

Given the non-Gaussian distribution of frequencies, Wilcoxon rank-sum tests were used to compare differences between the SSc patient group and the healthy donor group. All tests were two-sided, and differences were considered as significant at *p* < 0.05.

### Ethical approval

This study was conducted with the approval of the ethics committee of St. Louis Hospital (Paris, France).

## Results

### Opposite regulation of IL-13 production by TGF-β in T lymphocytes from healthy donors and patients with SSc

**Fig. 1:**
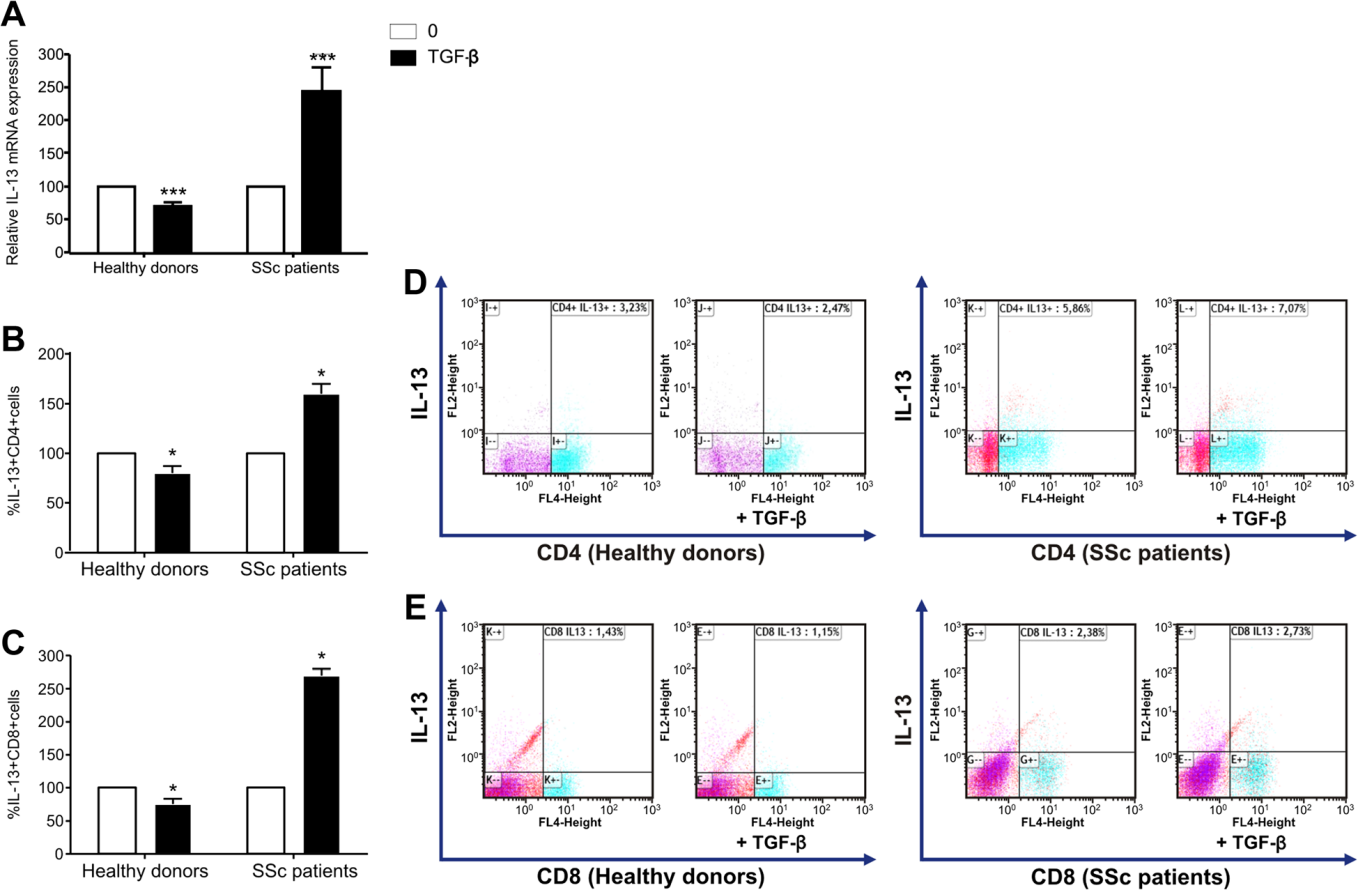
Effect of transforming growth factor (TGF)-β on interleukin (IL)-13 production by T lymphocytes from healthy donors and patients with SSc. Freshly isolated peripheral blood lymphocytes (PBLs) from healthy donors (n = 8) and patients with systemic sclerosis (SSc) (n = 10) were cultivated with anti-CD3/anti-CD28 antibodies and IL-2 for 5 days, then stimulated with phorbol 12-myristate 13-acetate (P) + ionomycin (I) for 5 h and treated or not with TGF-β for the last 4 h. **a** Levels of IL-13 mRNA measured by RT-PCR analysis and frequency of IL-13 cells in **b** and **d** CD4^+^ and **c** and **e** CD8^+^ T cells were quantified by intracellular staining by fluorescence-activated cell sorting (FACS) analysis. The results are presented as mean percentage (±SD) of IL-13 expression measured in the presence of TGF-β (*dark bars*) relative to the respective IL-13 expression detected in the absence of TGF-β (*white bars*). *p < 0.05; ***p < 0.001. Representative FACS histograms are from one representative healthy control (*left panel*) and one representative patient (*right panel*) showing double staining of **d** CD4^+^IL-13^+^ and **e** CD8^+^IL-13^+^ cells gated on CD3^+^ T lymphocytes, previously treated or not with TGF-β, as measured by intracellular IL-13 staining and surface expression of CD4 or CD8

The aim of the present study was to investigate the effect of TGF-β on IL-13 mRNA and protein expression in T lymphocytes from healthy donors and from patients with SSc. PBLs were incubated with TGF-β for 4 h before analysis. IL-13 mRNA production and IL-13 protein expression were measured by qRT-PCR and fluorescence-activated cell sorting (FACS), respectively. Mean (±SD) basal IL-13 mRNA levels were 0.94 ± 0.84 (n = 8) and 1.03 ± 0.82 (n = 10) in patients with SSc and in healthy controls, respectively. No significant difference was detected between the IL-13 baseline values in these two groups. As shown in Fig. 1a, TGF-β induced a significant decrease in IL-13 mRNA levels of PBLs from healthy donors, with a mean inhibition of 30 ± 10 % (n = 8; *p* < 0.001). The mean percentages of IL-13-producing CD4^+^ and CD8^+^ T cells in healthy donors reached 3.43 ± 0.6 % and 1.28 ± 0.4 %, respectively. TGF-β induced a significant decrease in the percentages of IL-13-producing CD4^+^ T cells (Fig. 1b) and IL-13-producing CD8^+^ T cells (Fig. 1c), with mean inhibition of 20 ± 7 % and 25 ± 8 % (n = 8; *p* < 0.05), respectively. In contrast (Fig. 1a), TGF-β induced a significant increase in IL-13 mRNA levels in PBLs from patients with SSc, with a mean fold increase of 2.4 ± 0.32 (n = 10, *p* < 0.05). Considering IL-13 protein levels in T-cell subsets from patients with SSc, the mean percentages of IL-13-producing CD4^+^ and CD8^+^ T cells were 3.86 ± 1.4 % and 1.3 ± 0.5 %, respectively. These levels were not significantly different from those detected in healthy donors, but TGF-β induced a significant increase in the percentages of IL-13-producing CD4^+^ T cells (Fig. 1b) and of IL-13-producing CD8^+^ T cells (Fig. 1c) from patients with SSc, with respective mean fold increases of 1.6 ± 0.05 and 2.7 ± 0.02 (n = 7; *p* < 0.05 and *p* < 0.001, respectively). Fig. 1d and Fig. 1e show FACS dot plots combining CD4^+^ or CD8^+^, respectively, and intracellular IL-13 staining for one representative healthy donor (*left panel*) and for one representative patient with SSc (*right panel*).

### Molecular mechanisms involved in IL-13 synthesis in response to TGF-β

**Fig. 2:**
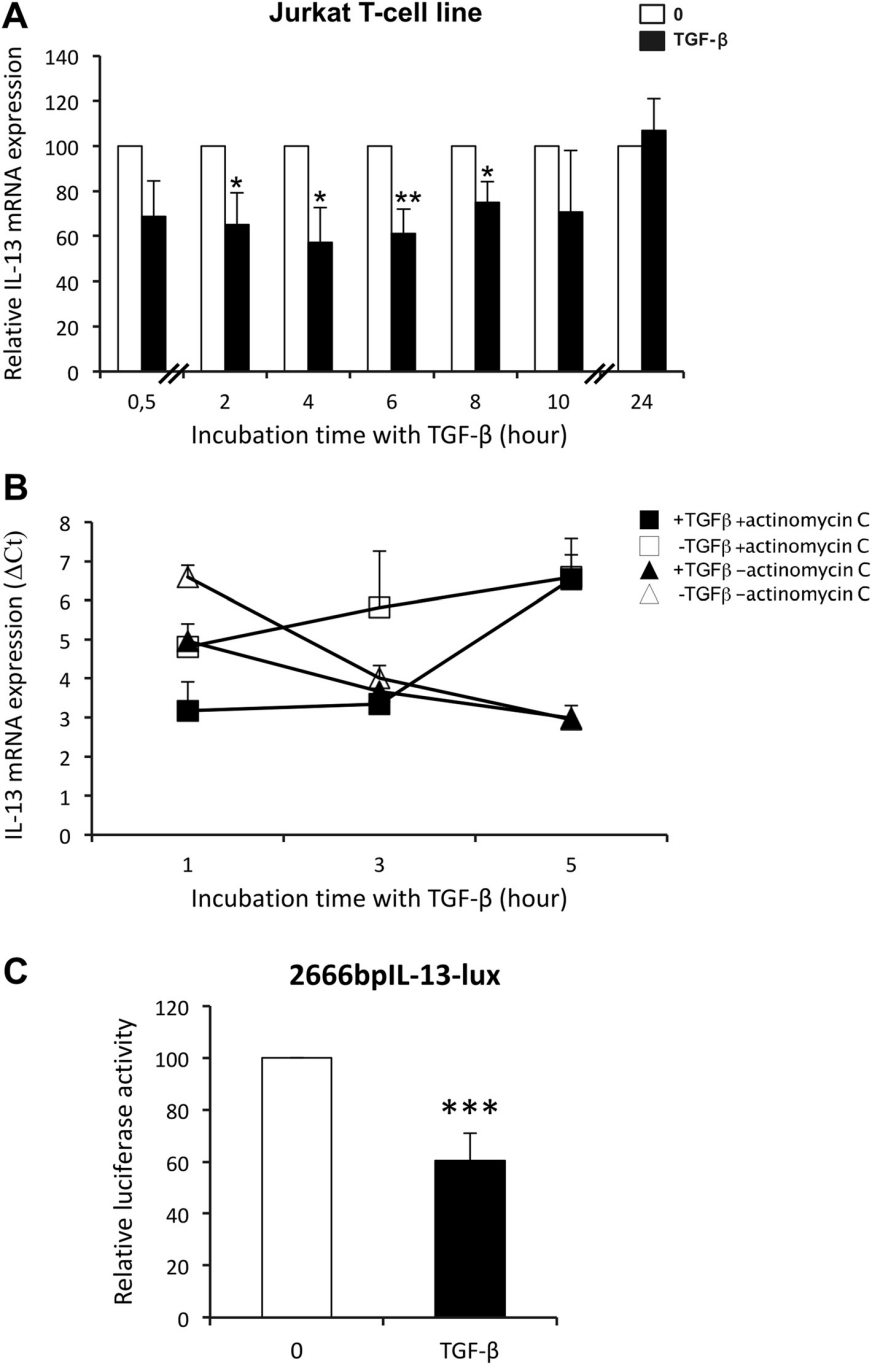
Transforming growth factor (TGF)-β modulates interleukin (IL)-13 mRNA steady-state levels by acting on IL-13 gene transcription. **a** Jurkat T cells were cultured in 0.5 % fetal calf serum (FCS)-containing medium for 16 h before addition of human recombinant TGF-β for the indicated time periods. IL-13 mRNA expression was measured by RT-PCR. The results are presented as mean percentage (±SD) of IL-13 expression measured in the presence of TGF-β (*dark bars*) relative to the respective IL-13 expression detected at the same time in the absence of TGF-β (*white bars*) and reported to 100 to allow comparison between time arrests. The results are representative of three independent experiments. *p < 0.05; **p < 0.01. **b** Following culture in 0.5 % FCS-containing medium for 16 h, Jurkat T cells were treated with (*dark dots*) and without (*white dots*) TGF-β for 4 h. In order to stop ongoing transcription, actinomycin D was then added (*squares*) or not (*triangles*) for a 1-, 3- and 5-h further incubation. IL-13 mRNA expression was analyzed by quantitative RT-PCR. The results are presented as comparative cycle threshold (∆C_t_), an arbitrary value from three independent experiments. **c** Jurkat T cells were transfected with pGL3 2666-bp IL-13 promoter construct/Luciferase (2666 bp-IL-13-Lux) promoter-containing plasmid. Cells were then treated (*dark bars*) or not (*white bars*) with TGF-β in 0.5 % FCS-containing medium for 24 h. The results are presented as mean percentage (±SD) of 2666 bp-IL13-Lux promoter activity detected in the presence of TGF-β relative to respective activity without TGF-β and reported to 100. The results are representative of six independent experiments. ***p < 0.001

To better understand the opposite IL-13 synthesis of T lymphocytes from healthy individuals and patients with SSc in response to TGF-β, the molecular pathways involved in TGF-β effects were dissected first in normal T lymphocytes and in Jurkat Th2 T-cell line. As observed in Fig. 2a, TGF-β also induced a rapid and transient significant decrease in IL-13 mRNA levels in Jurkat T cells. This decrease occurred without altering IL-13 mRNA stability (Fig. 2b). Thus, the cell line was chosen as a model of transfection assay for studying the effect of TGF-β on IL-13 production by normal T lymphocytes. To ascertain that TGF-β acts at the transcriptional level to inhibit IL-13 production, Jurkat T cells were transfected with an IL-13 promoter reporter construct (2666 bp-IL13-Lux). As presented in Fig. 2c, TGF-β exposure induced a significant decrease in IL-13 promoter luciferase activity compared with cells that were not exposed to TGF-β, with a mean inhibition of 40 ± 10 % (n = 6; *p* < 0.001). These data clearly indicate that TGF-β modulates IL-13 mRNA steady-state levels by acting on gene transcription.

### Involvement of Smad- and MAPK-dependent mechanisms

**Fig. 3:**
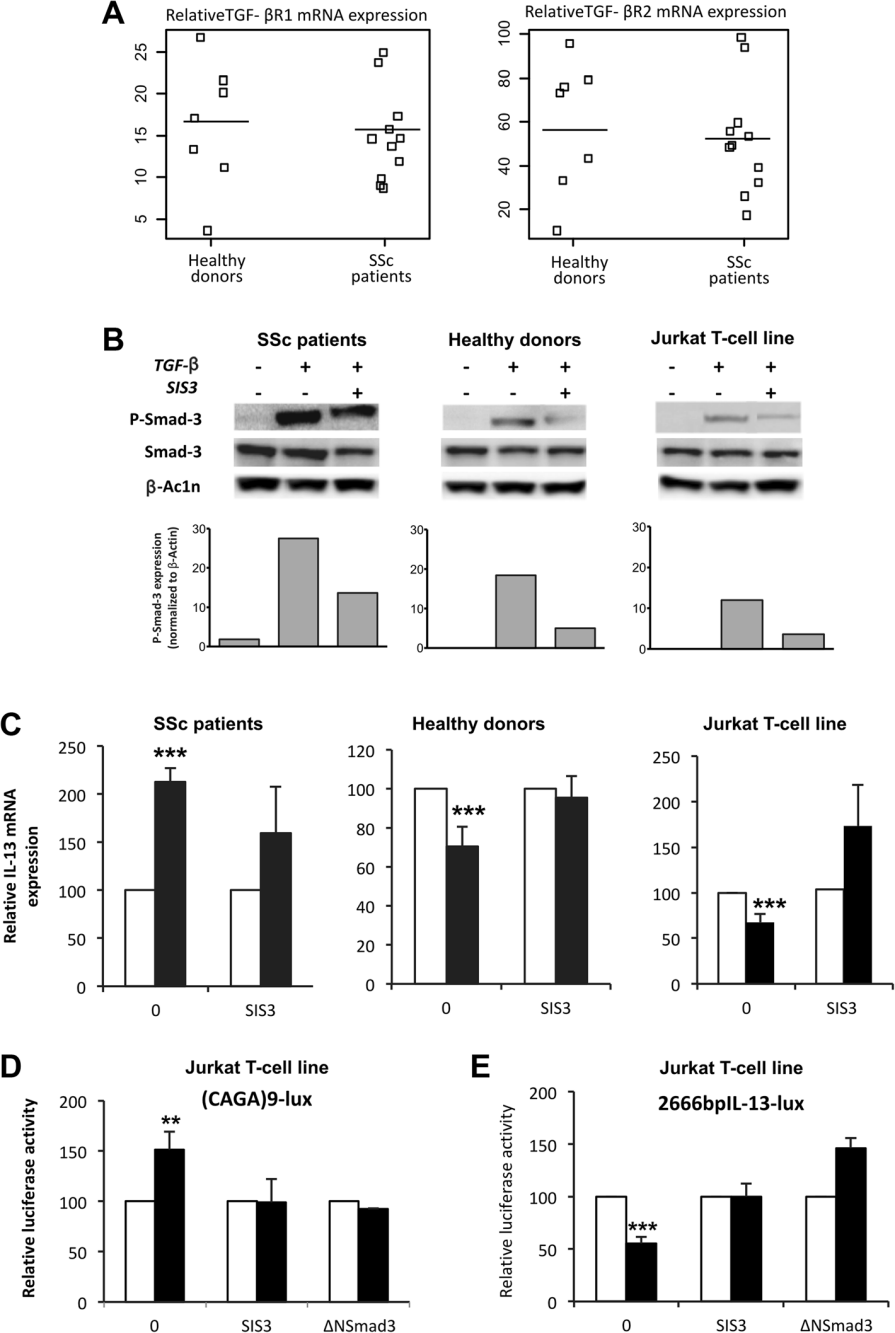
Transforming growth factor (TGF)-β increases interleukin (IL)-13 expression in the peripheral blood lymphocytes (PBLs) of patients with systemic sclerosis (SSc) via a Smad-dependent mechanism. **a** Type I TGF-β transmembrane receptor (TGF-βR1) and TGF-βR2 mRNA levels were measured by RT-PCR analysis in resting PBLs from healthy donors (n = 7) and patients with SSc (n = 11). **b** and **c** PBLs from patients with SSc (*left panel*) and healthy donors (*middle panel*) as Jurkat T cells (*right panel*) were cultured in 0.5 % fetal calf serum (FCS)-containing medium and treated with a specific Smad3 inhibitor (SIS3) for 1 h before addition of TGF-β or not for 4 h. **b** Expression of phosphorylated Smad3, nonphosphorylated Smad3 and β-actin was assessed by Western blotting. Semiquantitative analysis was performed by using ImageJ software, with β-actin levels used for normalization. Normalized data are schematized as *bars* under the Western blots. **c** Expression of IL-13 mRNA was measured by quantitative RT-PCR. The results are presented as mean percentage (±SD) of IL-13 expression detected in the presence of TGF-β (*dark bars*) relative to the respective IL-13 expression without TGF-β (*white bars*) and reported to 100 to allow comparison. **d** and **e** Jurkat T cells were transfected with **d** (CAGA)9-Lux or **e** pGL3 2666-bp IL-13 promoter construct/Luciferase (2666 bp-IL13-Lux) . Cells were then treated (*dark bars*) or not (*white bars*) with TGF-β in 0.5 % FCS-containing medium for 24 h. Jurkat T cells transfected with (CAGA)9-Lux **d** or 2666 bp-IL13-Lux promoter (**e**) were treated with or without SIS3 1 h before adding (or not) TGF-β (*middle double bars*). Jurkat T cells were cotransfected with **c** (CAGA)9-Lux promoter and a dominant negative of Smad3 (∆NSmad3) or **e** 2666 bp-IL13-Lux promoter and ∆NSmad3, and then Jurkat cells were treated with or without TGF-β (*right double bars*). The results are presented as mean percentage (±SD) of (CAGA)9-Lux or 2666 bp-IL13-Lux promoter activity detected in the presence of TGF-β (*dark bars*) relative to respective activity without TGF-β (*white bars*) and reported to 100 to allow comparison. **b**–**e** Results are representative of four independent experiments. **p < 0.01; ***p < 0.001

The mechanisms by which TGF-β alters IL-13 synthesis could involve either TGF-β receptor expression or several signaling pathways, including Smad and MAPK. First, the differences in responses of T lymphocytes from healthy donors and patients with SSc were not linked to an alteration in TGF-β receptor level expression, because TGFβRI and TGFβRII mRNA expression in resting PBLs from healthy donors and patients with SSc was not significantly different (Fig. 3a), nor was the membrane expression detected by FACS analysis (data not shown). In regard to Smad pathway activation, TGF-β induced a rapid and sustained phosphorylation of Smad3 molecules in T lymphocytes from patients with SSc that was more intense than observed in T lymphocytes from healthy donors and in Jurkat T cells, although with no significant difference when we considered the nine patients with SSc (Fig. 3b). The TGF-β–induced Smad3 phosphorylation in PBLs from patients with SSc (Fig. 3b, *left panel*) was decreased in the presence of a specific Smad3 inhibitor (SIS3), although to a lesser extent than the decrease observed in T lymphocytes from healthy donors and in Jurkat T cells (Fig. 3b, *middle* and *right panels*, respectively). Moreover, the increase in IL-13 mRNA levels induced by TGF-β observed in patients with SSc (Fig. 3c, *left panel*) was partially reversed in the presence of SIS3, and, conversely, the decrease in IL-13 mRNA levels induced by TGF-β in PBLs from healthy donors and Jurkat T cells was totally reversed (Fig. 3c, *right panels*).

The ability of TGF-β to activate the Smad3/4 transcriptional activity was assessed by transfection assay of the Jurkat cell line with the Smad3/4-specific reporter construct ([CAGA]9-Lux). As shown in Fig. 3d, TGF-β induced a significant mean fold increase of 1.54 ± 0.17 (n = 4; *p* < 0.01) in transactivation of the (CAGA)9-Lux promoter after 24 h of exposure. This was reversed by cotransfection of the (CAGA)9-Lux promoter with a dominant negative of Smad3 (∆NSmad3) or the addition of SIS3 (Fig. 3d). Of interest, addition of SIS3 or overexpression of ∆NSmad3 totally reversed TGF-β-induced inhibition of IL-13 promoter activity in Jurkat T cells (Fig. 3e), clearly demonstrating involvement of the Smad pathway in TGF-β-induced modulation of IL-13 expression in T cells.

Altogether, these results indicate that the TGF-β-induced Smad pathway is involved in stimulation of IL-13 expression by TGF-β in T lymphocytes from patients with SSc and, according to the effect of SIS3, might be more activable in T lymphocytes from patients with SSc than from healthy donors.

**Fig. 4:**
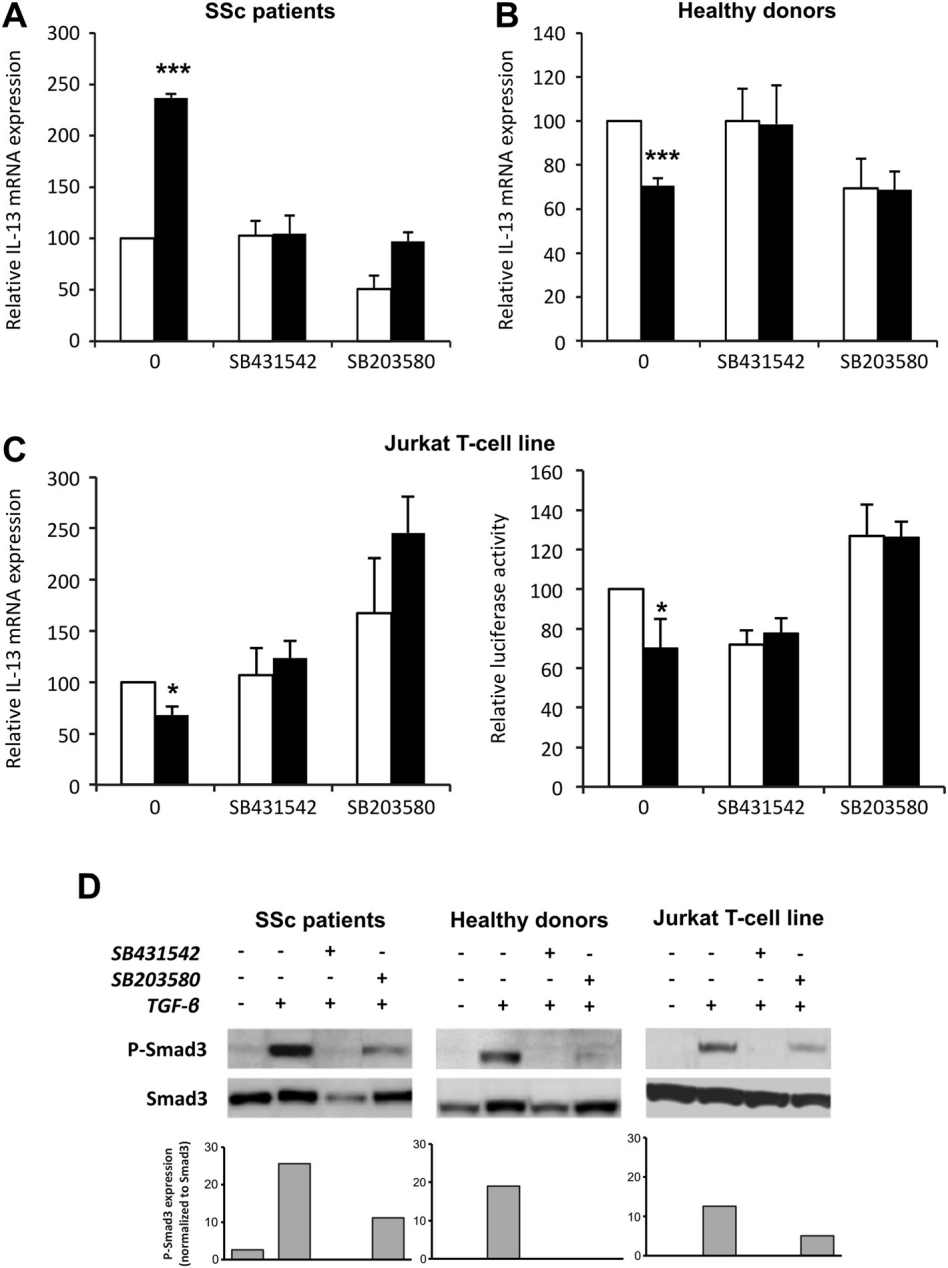
Implication of the p38 mitogen-activated protein kinase pathway downstream of transforming growth factor (TGF)-β receptors on peripheral blood lymphocytes (PBLs). PBLs from **a** patients with SSc and **b** healthy donors were treated with (or without) SB431542 (activin receptor-like kinase ALK5 inhibitor) or SB203580 (specific p38 inhibitor) for 1 h before the adding, or not, TGF-β. Interleukin (IL)-13 mRNA expression was measured by RT-PCR. The results are presented as mean percentage (±SD) of IL-13 expression detected in the presence (*dark bars*) or absence (*white bars*) of TGF-β relative to basal IL-13 expression taken as 100. **c** After 16 h of culture in 0.5 % fetal calf serum (FCS)-containing medium, Jurkat T cells were treated with (or without) SB431542 or SB203580 for 1 h before adding, or not, TGF-β, and IL-13 mRNA expression was measured by RT-PCR (*left panel*). Jurkat T cells were transfected with pGL3 2666-bp IL-13 promoter construct/Luciferase (2666 bp-IL13-Lux) or empty pGL3-Lux promoter and then treated or not with SB431542 or SB203580 for 1 h before adding TGF-β (*dark bars*) or not (*white bars*) for a further 24-h culture in 0.5 % FCS-containing medium (*right panel*). The results are presented as mean percentage (±SD) of IL-13 expression (*left panel*) or 2666 bp-IL13-Lux promoter activity (*right panel*) detected in the presence (*dark bars*) or absence (*white bars*) of TGF-β relative to basal IL-13 expression taken as 100. The results shown in **a**–**c** are representative of four independent experiments. *p < 0.05; ***p < 0.001. **d** Phosphorylated Smad3 and respective nonphosphorylated protein expression was detected by Western blotting in PBLs from patients with SSc (*left panel*), healthy donors (*middle panel*) and Jurkat T cells (*right panel*) cultured in 0.5 % FCS-containing medium with SB431542 or SB203580 for 1 h before addition of TGF-β or not for 4 h. The results are representative of four independent experiments. Semiquantitative analysis of blots was performed by using ImageJ software with Smad3 levels for normalization. Normalized data are schematized as *bars* under the Western blots

The p38 pathway regulates several Th2 cytokines including IL-13 [20] and the activation of p38 pathway downstream of TGF-β receptors can potentially regulate GATA-3/IL-13 expression [21]. Our results show that TGF-β did activate the p38 pathway in T cells because TGF-β induced a rapid and prolonged phosphorylation of p38 (data not shown). The role of the p38 pathway in TGF-β-induced IL-13 synthesis was investigated by using a specific p38 inhibitor (SB203580). As shown in Fig. 4a, both the addition of either an ALK5 inhibitor (SB431542) or SB203580 reversed the increased level of IL-13 mRNA transcripts detected in the presence of TGF-β in PBLs from patients with SSc. Both inhibitors also reversed the inhibitory effect of TGF-β on IL-13 mRNA expression in healthy donors (Fig. 4b), as well as in the Jurkat cell line (Fig. 4c, *left panel*). The role of the p38 pathway in IL-13 gene expression was investigated using transfected Jurkat T cells, and our results show that the addition of SB431542 or SB203580 reversed the effect of TGF-β on IL-13 promoter activity, as well as on IL-13 mRNA transcripts (Fig. 4c, *right panel*).

The effects of SB431542 and SB203580 on Smad pathway activation were studied in PBLs from patients with SSc by Western blot analysis. As expected, the ALK5 inhibitor SB431542 totally abolished TGF-β-induced Smad3 phosphorylation in PBLs from patients with SSc and healthy donors, as well as in Jurkat T cells (Fig. 4d). Interestingly, the p38 inhibitor partially decreased TGF-β-induced Smad3 phosphorylation in SSc T cells, whereas it totally inhibited Smad3 phosphorylation in healthy donors (Fig. 4d).

Altogether, these results demonstrate a cooperative role between Smad and p38-MAPK signaling in mediating TGF-β-induced upregulation of IL-13 expression in T lymphocytes from patients with SSc.

### Upregulation of IL-13 gene expression by TGF-β occurs via GATA-3 transcription factor regulation: implication of Smad3 and MAPK signaling pathways

**Fig. 5:**
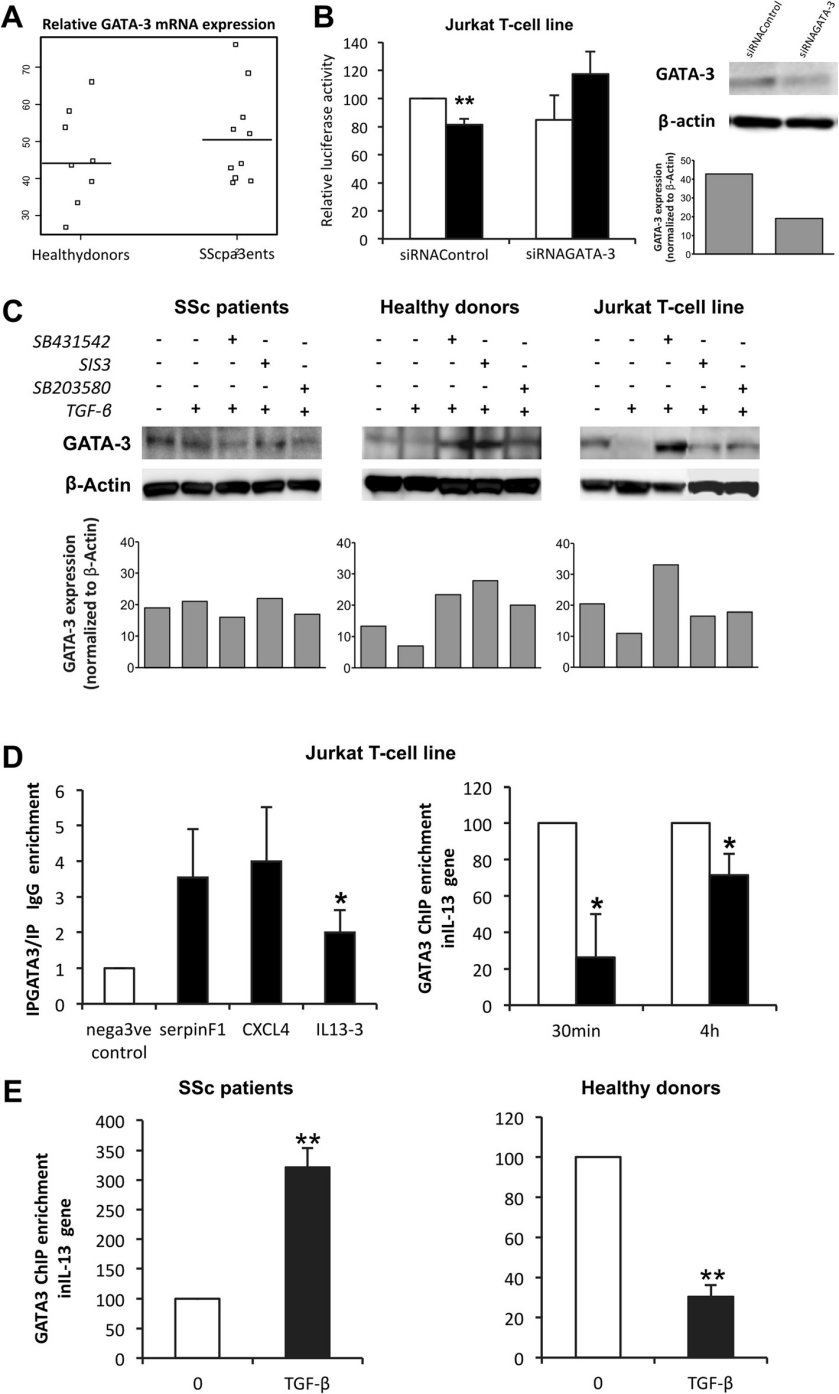
Upregulation of interleukin (IL)-13 gene expression by transforming growth factor (TGF)-β occurs via GATA-3 transcription factor regulation: implication of Smad3 and mitogen-activated protein kinase (MAPK) signaling pathways. **a** GATA-3 mRNA levels in peripheral blood lymphocytes (PBLs) from patients with systemic sclerosis (SSc) (n = 11) and healthy donors (n = 8) were measured by RT-PCR analysis. **b** Jurkat T cells were cotransfected with pGL3 2666-bp IL-13 promoter construct/Luciferase (2666 bp-IL13-Lux) promoter and either a small interfering RNA (siRNA) specific for GATA-3 or siRNA control. Jurkat transfected cells were then treated or not with TGF-β for 24 h in 0.5 % fetal calf serum (FCS)-containing medium. The results are presented as mean percentage (±SD) of 2666 bp-IL13-Lux promoter activity in the presence (*dark bars*) or absence (*white bars*) of TGF-β relative to control siRNA activity without TGF-β taken as 100 (*left panel*). Expression of GATA-3 and β-actin in total lysates from control or GATA-3 siRNA was measured by Western blotting (*right panel*). The results are representative of three independent experiments. **p < 0.01. **c** Expression of GATA-3 and β-actin was measured by Western blotting in nuclear lysates from patients with SSc (*left panel*), healthy donors (*middle panel*) and Jurkat T cells (*right panel*) cultured in 0.5 % FCS-containing medium with SB431542 (activin receptor-like kinase ALK5 inhibitor), specific Smad3 inhibitor (SIS3) or SB203580 (specific p38 inhibitor) for 1 h before addition of TGF-β for 4 h. The results are representative of four independent experiments. Semiquantitative analysis of the blots was performed by using ImageJ software with β-actin levels for normalization. Normalized data are schematized as *bars* under the Western blots. **d** Jurkat T cells were cultured in 0.5 % FCS- containing medium for 16 h before addition of human recombinant TGF-β for 4 h (*left panel*) or for 30 min or 4 h (*right panel*). **e** PBLs from patients with SSc (n = 3) (*left panel*) and healthy donors (n = 3) (*right panel*) were stimulated with anti-CD3/anti-CD28 antibodies and IL-2 for 5 days and then treated or not with TGF-β for the last 4 h. **d** and **e** After precipitation of the protein–DNA complexes with specific antibody to GATA-3 or control immunoglobulin G (IgG), PCR amplification of the IL-13 fragment was performed using IL-13 promoter primers. GATA-3 chromatin immunoprecipitation (ChIP) enrichment = ChIP/input × 100 was determined by quantitative RT-PCR. The results are presented as mean (±SD) GATA-3 ChIP enrichment (GATA-3/IgG ratio) in the IL-13 gene after 4-h incubation with TGF-β (*dark bars*) and relative to respective expression in the absence of TGF-β (*white bars*) and reported to 100 to allow comparison of enrichment. The amplified *SERPINF1* and *CXCL4* promoter regions were used as positive controls for GATA-3 binding in Jurkat T-cells. *p < 0.05, **p < 0.01

Because GATA-3 has been shown to activate both mouse [44] and human [45] IL-13 promoters, respectively, in splenic T lymphocytes and Jurkat T cells, and because TGF-β was shown to inhibit Th2 differentiation via repression of the transcription factor GATA-3 [24], we hypothesized that GATA-3 could be differentially modulated by TGF-β in PBLs from patients with SSc and healthy donors and could be responsible for the effects of TGF-β on IL-13 expression. Basal GATA-3 mRNA levels were compared in PBLs from patients with SSc and healthy donors, and no differences were observed (Fig. 5a). To determine whether GATA-3 was involved in TGF-β-induced modulation of IL-13 expression, Jurkat T cells were transfected with a small interfering RNA (siRNA), which specifically targeted GATA-3 mRNA and specifically downregulated GATA-3 protein expression, as shown in Fig. 5b (*right panel*). Cotransfection of Jurkat T cells with 2666 bp-IL13-Lux plasmid and GATA-3 siRNA induced a significant inhibition of IL-13 promoter activity and reversed the inhibitory effect of TGF-β. This clearly indicates that GATA-3 is involved in both IL-13 synthesis and TGF-β inhibitory effects. To determine whether TGF-β had a specific, direct effect on GATA-3 expression in T cells, GATA-3 nuclear protein expression after incubation with TGF-β was assessed by Western blot analysis. As shown in Fig. 5c, GATA-3 expression was not modulated in PBLs from patients with SSc (*left panel*), whereas it was decreased in response to TGF-β in healthy donors (*middle panel*) and Jurkat T cells (*right panel*). The role of both the Smad and MAPK pathways in GATA-3 expression was investigated, and the results shown in Fig. 5c indicated that SB431542, SIS3 and SB203580 did not affect GATA-3 expression in PBLs from patients with SSc (*left panel*). In contrast, these inhibitors reversed the TGF-β effect on GATA-3 inhibition in PBLs from healthy donors and in Jurkat T cells with even an increase after ALK5 inhibitor addition (*middle* and *right panels*). This could suggest that GATA-3 nuclear translocation might be controlled by Smad-dependent factors.

To assess the involvement of the GATA-3 transcription factor in the regulation of the IL-13 gene by TGF-β, chromatin was extracted from T cells and immunoprecipitated with anti-GATA-3 antibody. PCR amplification revealed GATA-3 enrichment in the IL-13 promoter gene by chromatin immunoprecipitation (ChIP) as compared with a negative *KRT1* promoter gene control and two positive genes, *SERPINF1* and *CXCL4*, in PBLs and Jurkat T cells (Fig. 5d, *left panel*). The effect of TGF-β on the capacity of GATA-3 to bind to the IL-13 promoter was investigated after cell treatment with TGF-β for 4 h. As shown in Fig. 5d (*right panel*), TGF-β decreased GATA-3 ChIP enrichment of IL-13 gene in Jurkat T cells by 74 ± 24 % and 29 ± 12 % after 30 min and 4 h of treatment, respectively.

Furthermore, our data show that TGF-β increased GATA-3 ChIP enrichment of the IL-13 gene by 336 ± 70 % (n = 3; *p* < 0.01) in PBLs from patients with SSc (Fig. 5e, *left panel*), whereas TGF-β decreased GATA-3 ChIP enrichment of the IL-13 gene by 70 ± 6 % (n = 3; *p* < 0.01) in PBLs from healthy donors (Fig. 5e, *right panel*).

Overall, GATA-3 binding capacity on IL-13 promoter was increased in patients with SSc and decreased in healthy donors. These results demonstrated that the absence of GATA-3 downregulation by TGF-β, and conversely its increase, explained the overexpression of IL-13 expression in patients with SSc.

## Discussion

The pathogenesis of SSc is not completely elucidated and includes abnormal immune system activation; however, the pathways active in the effector mechanisms are still not completely understood. As TGF-β and IL-13 have been depicted as key mediators in the pathogenesis of SSc, our concern in the present study was to assess the precise role of TGF-β in IL-13 expression in T lymphocytes of patients with SSc. The present study elucidates a new aspect of the pathogenic role of TGF-β in SSc by showing upregulation of IL-13 synthesis in response to TGF-β in the peripheral CD4^+^ and CD8^+^ T cells of patients with SSc, whereas IL-13 expression was downregulated by TGF-β in healthy donors.

Higher IL-13 production by peripheral blood [36] and skin biopsy [10] CD8^+^ T cells had previously shown that SSc is associated with IL-13 dysregulation. Accordingly, our results allowed us to demonstrate for the first time a relationship between increase IL-13 production in CD4^+^ and CD8^+^ T cells and TGF-β signaling in patients with SSc, and they enabled us to further identify mechanisms involved in this dysregulation.

In spite of the fact that TGF-β is a key mediator in the pathological tissue fibrosis in many diseases [23], TGF-β is also known as a potent physiological immunomodulator in mammals that is able to inhibit Th2 and Th1 differentiation by repressing activation of GATA-3 and T-bet transcription factors, respectively [23]. GATA-3 has been recognized as the main transcription factor regulating IL-13 gene expression in T cells [35, 38], and it has also been associated with IL-13 overproduction in patients with SSc [40]. The present study did not allow us to set up GATA-3 as a potent biomarker of immune dysfunction in SSc, as previously described by Medsger et al. [40]. Our results show that TGF-β was able to regulate GATA-3 ChIP enrichment of the IL-13 gene, resulting in the respective stimulation or inhibition of IL-13 expression in patients with SSc and in healthy donors. This is in contrast to the findings of Medsger and colleagues, who described that IL-13 overproduction by naïve CD8^+^ T cells from patients with SSc was related to GATA-3 dysregulation [40], whereas we detected similar levels of GATA-3 mRNA expression in patients with SSc and healthy donors. This difference may be explained by the use of whole T-cell populations in our study, whereas Medsger et al. used isolated CD8^+^ T cells.

However, the mechanism involved in GATA-3 dysregulation remained to be elucidated. Our results show that IL-13 gene regulation by TGF-β occurs via Smad- and p38-dependent pathways and that both of these pathways are involved in GATA-3 modulation. The involvement of the Smad pathway was demonstrated by using the specific SIS3 inhibitor, which reversed the TGF-β effects on IL-13 and GATA-3 expression. In accordance with our results, in vivo studies demonstrated that Smad3-deficient mice exhibited increased IL-13 cytokine levels in association with an upregulation of GATA-3 levels, suggesting that GATA-3 depends on Smad3 expression for regulation of IL-13 synthesis [21]. In addition, GATA-3 has been shown to physically and functionally interact with Smad3 to allow TGF-β regulation of GATA-3 target genes [44]. GATA-3-mediated activation of several cytokine promoters, including IL-4 and IL-5, could be blocked by a zinc finger protein named “friend of GATA” (FOG), which acts as a GATA-3 repressor in naïve Th cells, and its downregulation is key for Th2 cell development [45]. Functional interactions between FOG and GATA-3 mechanisms have not been described, but FOG may interact with a specific corepressor, mCtBP2 [46], suggesting that its downregulation might favor upregulation of IL-13 expression in T cells of patients with SSc. We also demonstrated that the cross-talk between MAPKs and Smad proteins modulates TGF-β effects on T cells from patients with SSc. Indeed, our data show that p38 inhibition reversed the effects of TGF-β on GATA-3 and IL-13 expression and decreased TGF-β-induced phosphorylation of Smad3. The role of the p38-MAPK pathway in regulating GATA-3 phosphorylation and Th2 cytokine gene expression has been highlighted in two previous studies, evidencing that GATA-3 phosphorylation by p38-MAPK facilitates nuclear translocation and allows regulation of the IL-5, IL-4 and IL-13 genes [47, 48].

**Fig. 6:**
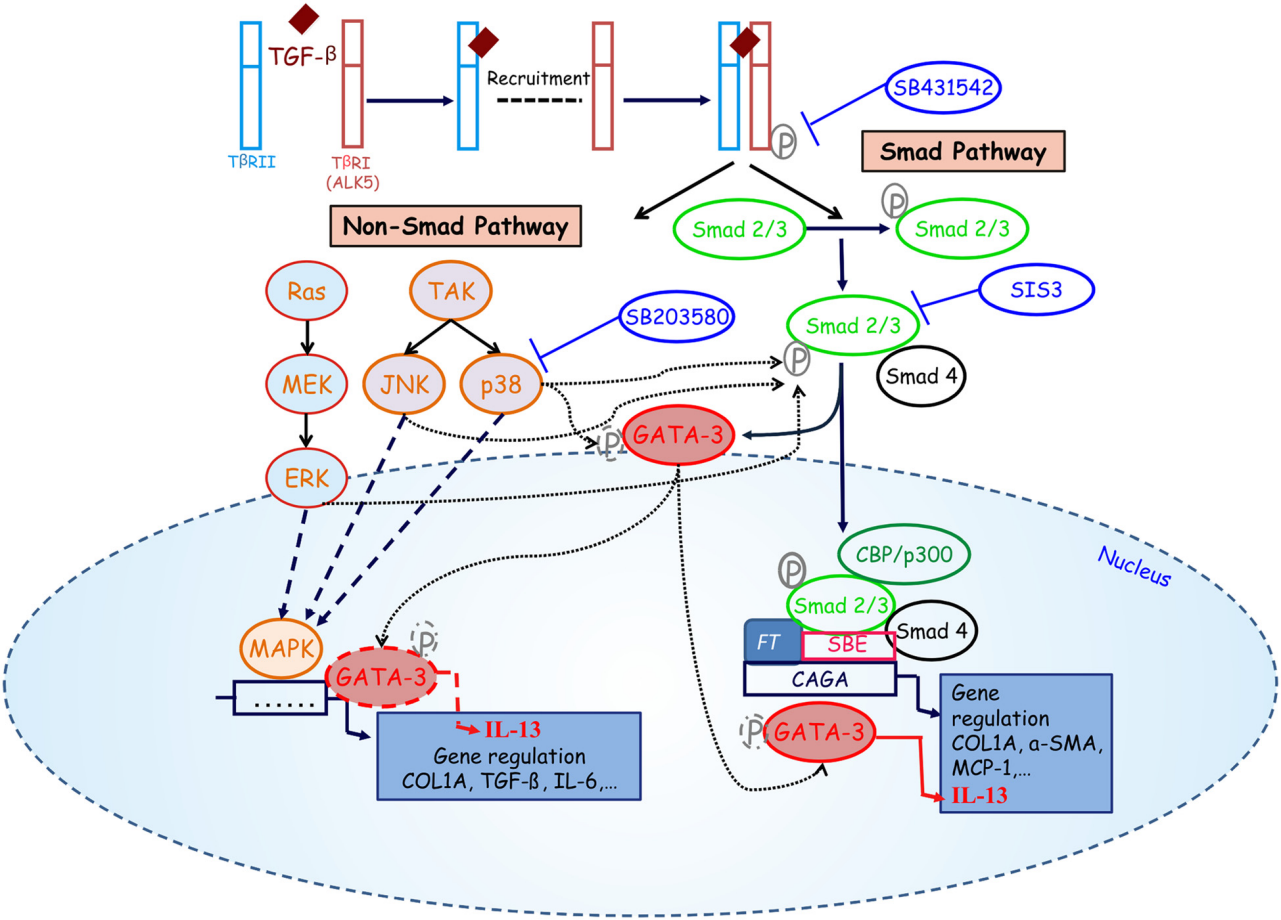
Schematic hypothesis about interleukin (IL)-13 synthesis induced through Smad and mitogen-activated protein kinase (MAPK) pathways in response to transforming growth factor (TGF)-β in T cells of patients with systemic sclerosis. TGF-β binds first to its type II receptor (TβRII), which then recruits and phosphorylates the type I receptor (TβRI), leading to activin receptor-like kinase (ALK5) activation within the receptor complex. The TβRI–TβRII complex then phosphorylates Smad 2/3 proteins, which complex with Smad4 to accumulate in the nucleus and act as transcription factors of target genes. In the non-Smad pathway, the TβRI–TβRII complex transmits its signal through other factors, such as by the Ras-extracellular signal-regulated kinase (Ras-ERK) pathway, TGF-β-activated kinase (TAK), c-Jun N-terminal kinase (JNK) and p38-MAPK pathways. Under activation, these factors translocate to the nucleus and regulate the expression of target genes. We observed that the upregulated IL-13 expression induced by TGF-β occurs via GATA-3 transcription factor modulation through Smad3 and p38-MAPK signaling pathways, which is reversed by the specific Smad3 inhibitor SIS3 or by the specific p38 inhibitor SB203580, respectively. Additionally, the ALK5-specific inhibition by SB431542 abolishes the TGF-β-induced Smad3 phosphorylation. *CBP* CREB-binding protein, *COL1A* collagen type I alpha, *MCP-1* monocyte chemoattractant protein 1, *SBE* Smad-binding element, *α-SMA* α-smooth muscle actin

To schematize the results of our present work, we propose a mechanistic scheme with signaling pathways involved in TGF-β-induced IL-13 synthesis in T lymphocytes of patients with SSc (Fig. 6).

Multiple factors, including cellular interactions, T-cell receptor signals, cytokines and transcription factors, could cooperate with TGF-β to influence the IL-13 response in patients with SSc. In particular, high levels of IL-4 and TNF-α observed in patients with SSc could be responsible for stimulation of the GATA-3 [49] or Smad [50] pathway, respectively. In addition, T-bet transcription factor polymorphisms have been described in SSc [51]. As this factor is able to regulate IL-13 synthesis via GATA-3 repression in healthy individuals [52], its alteration could be involved in the upregulation of GATA-3 observed in the IL-13 promoter in T cells of patients with SSc. In this context, recent studies of GATA-3 as a therapeutic target in allergic and inflammatory diseases are being developed, including various animal models and clinical applications. The GATA-3-specific inhibitor DNAzyme hgd40 is already being tested in clinical trials (www.clinicaltrials.gov) to treat allergic asthma (NCT01743768) and ulcerative colitis (NCT02129439). It could thus be readily available for trials in patients with SSc after any antifibrotic potential of this new specific inhibitor has been confirmed in animal models of SSc.

## Conclusions

In summary, our results provide new evidence about the active contribution of Smad and p38 pathways downstream to TGF-β, leading to regulation of GATA-3 functions and stimulation of IL-13 synthesis in T lymphocytes of patients with SSc. GATA-3 might be considered as a potential target for new therapies, and prevention of its interaction with other factors by inhibiting p38-MAPK or Smad3 pathways may provide a new approach for the treatment of SSc.

## Abbreviations

ALK: Activin receptor-like kinase
APC: Allophycocyanin
ChIP: Chromatin immunoprecipitation
CMV: Cytomegalovirus
C_t_: Comparative cycle threshold
ECM: Extracellular matrix
EDTA: Ethylenediaminetetraacetic acid
ERK: Extracellular signal-regulated kinase
FACS: Fluorescence-activated cell sorting
FCS: Fetal calf serum
FITC: Fluorescein isothiocyanate
FOG: Friend of GATA
GUS: β-glucuronidase
HEPES: N-2-hydroxyethylpiperazine-N-2-ethanesulfonic acid
IgG: Immunoglobulin G
IL: Interleukin
IL-13Rα2: Interleukin-13 receptor α-chain 2
JNK: c-Jun N-terminal kinase
MAPK: Mitogen-activated protein kinase
MMP: Matrix metalloproteinase
NP-40: Nonidet P-40
PBL: Peripheral blood lymphocyte
PBMC: Peripheral blood mononuclear cell
PBS: Phosphate-buffered saline
PE: Phycoerythrin
qRT-PCR: Quantitative real-time reverse transcription polymerase chain reaction
siRNA: Small interfering RNA
SSc: Systemic sclerosis
TAK: Transforming growth factor-β-activated kinase
TE: Tris-ethylenediaminetetraacetic acid
TGF-β: Transforming growth factor-β
TGFβR: Transforming growth factor-β transmembrane receptor
Th2: Type 2 helper T cell
